# Supplementary material for: Evaluation of Conserved RNA Secondary Structures within and between Geographic Lineages of Zika Virus
Source: Life (Basel). 2021 Apr 14;11(4):344. doi: 10.3390/life11040344 (PMC8070784; doi:10.3390/life11040344)
Supplement: Supplementary file 1 [file life-11-00344-s001.pdf]

life

MDPI

Supplementary Material

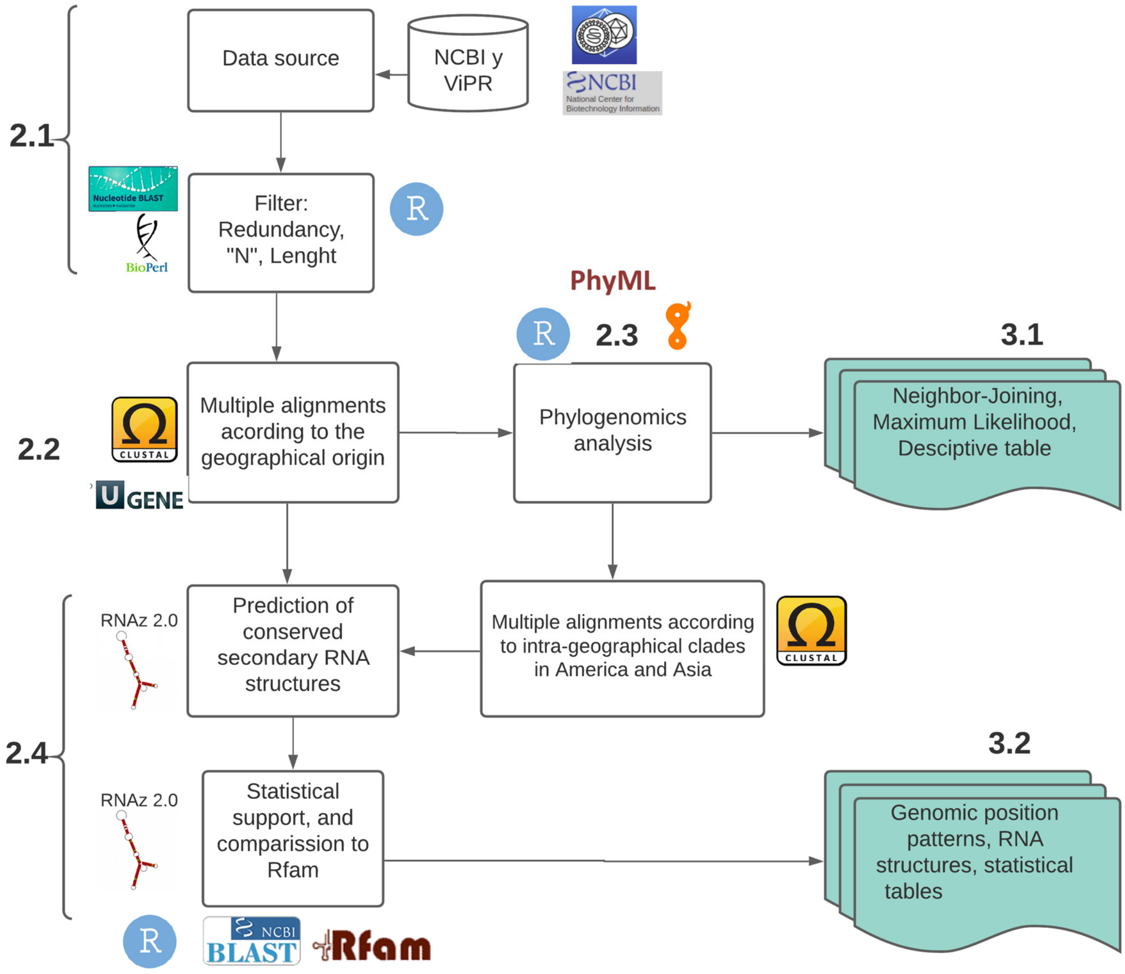

Figure S1. Graphical abstract of materials, methods and results sections.

A B

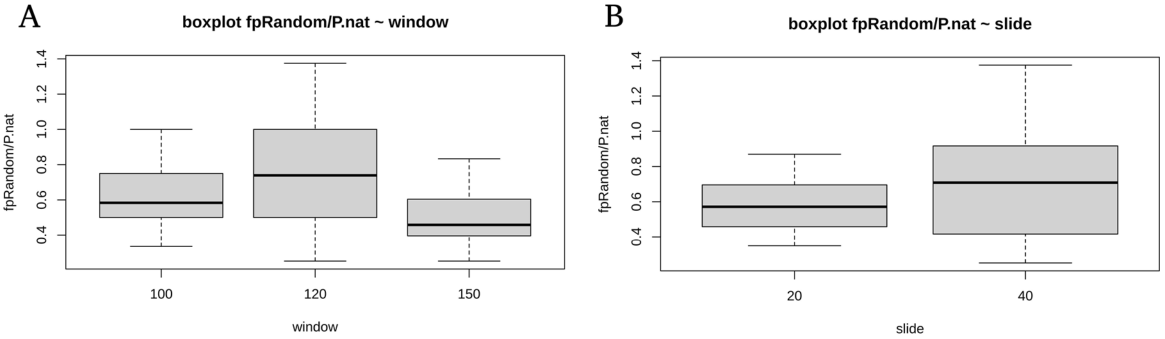

C

factor	DF	Sum sq	Mean sq	F Value	Pr(>F)	Significance
Window	2	0.2448	0.12242	5.383	0.00738	**
Slide	1	0.0380	0.03801	1.671	0.20157	/
Window:Slide	2	0.0375	0.01874	0.824	0.44406	/
Residuals	54	1.2280	0.02274	NA	NA	

D

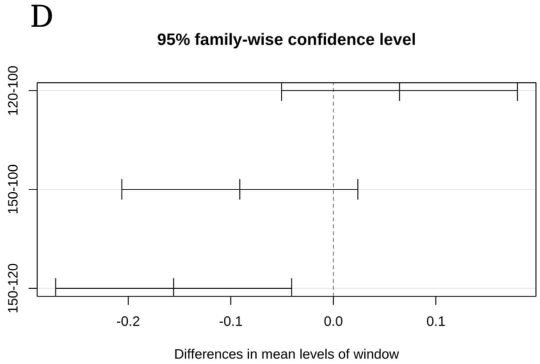

Figure S2. Results of the two-way ANOVA, comparing the window size factor (100, 120 and 150), and the sliding size factor (20.40). **(A)** Boxplot of the window size factor. **(B)** Boxplot of the slip size factor **(C)** Output two-way ANOVA. **(D)** Tukey plot of the window size factor. Statistical assumptions: Normality Shapiro test (p value = 0.0533), Bartlett homoscedasticity test (Window p value = 0.1674, Slide p value = 0.02).

Table S1. Statistical evaluation for complete alignments of geographic lineages. (%FP = False positives rate).

Region	% FP	Specificity (%)	N° Windows (n)
Global	0.003	99.65	53100
Africa	0.005	99.49	53100
Asia	0.019	98.12	53100
America	0.023	97.61	53100
Oceania	0.034	96.60	53100
Asi_SE	0.021	97.87	53100
Asi_cont	0.030	96.96	53100
Bra_Cl	0.032	96.83	53100
Car_Cl	0.032	96.85	53100
Col_Cl	0.034	96.60	53100
Mex_Cl	0.033	96.70	53100

Table S2. Statistical evaluation in the randomized alignments performed by each window detected as positive for structural RNA at <u>inter</u>-geographic lineages level. (L = Locus, W = Window, %FP = False positives rate).

Region	L	W	%Fp	Region	L	W	%Fp	Region	L	W	%Fp	Region	L	W	%Fp	Region	L	W	%Fp
Global	1	1	0.010	Africa	1	9	0.000	America	1	15	0.025	Asia	1	12	0.030	Oceania	1	12	0.035
Global	1	2	0.000	Africa	1	10	0.005	America	1	16	0.025	Asia	1	13	0.015	Oceania	1	14	0.030
Global	1	3	0.000	Africa	1	11	0.000	America	2	17	0.030	Asia	2	14	0.025	Oceania	1	15	0.060
Global	1	4	0.000	Africa	2	12	0.010	America	3	18	0.020	Asia	3	15	0.020	Oceania	1	16	0.030
Global	1	5	0.000	Africa	3	13	0.015	America	4	19	0.025	Asia	4	16	0.040	Oceania	2	18	0.045

Global	1	6	0.000	Africa	4	14	0.010	America	6	22	0.030	Asia	5	17	0.030	Oceania	4	21	0.035
Global	1	7	0.005	Africa	4	15	0.005	America	8	26	0.030	Asia	6	19	0.040	Oceania	5	23	0.035
Global	1	8	0.005	America	1	1	0.000	America	9	27	0.015	Asia	8	22	0.010	Oceania	5	24	0.035
Global	1	9	0.000	America	1	2	0.000	America	9	28	0.015	Asia	8	23	0.025	Oceania	5	25	0.055
Global	1	10	0.005	America	1	3	0.005	America	9	29	0.010	Asia	9	24	0.045	Oceania	5	26	0.060
Global	1	11	0.000	America	1	4	0.015	Asia	1	1	0.000	Oceania	1	1	0.010	Oceania	5	29	0.025
Global	1	12	0.000	America	1	5	0.050	Asia	1	2	0.025	Oceania	1	2	0.020	Oceania	6	31	0.040
Global	2	13	0.005	America	1	6	0.085	Asia	1	3	0.005	Oceania	1	3	0.035	Oceania	8	36	0.030
Africa	1	1	0.005	America	1	7	0.045	Asia	1	4	0.020	Oceania	1	4	0.035	Oceania	10	40	0.045
Africa	1	2	0.005	America	1	8	0.020	Asia	1	5	0.030	Oceania	1	5	0.030	Oceania	13	46	0.025
Africa	1	3	0.010	America	1	9	0.030	Asia	1	6	0.005	Oceania	1	6	0.065	Oceania	13	47	0.020
Africa	1	4	0.025	America	1	10	0.035	Asia	1	7	0.025	Oceania	1	7	0.020	Oceania	13	48	0.035
Africa	1	5	0.045	America	1	11	0.045	Asia	1	8	0.015	Oceania	1	8	0.020	/	/	/	/
Africa	1	6	0.035	America	1	12	0.015	Asia	1	9	0.015	Oceania	1	9	0.010	/	/	/	/
Africa	1	7	0.015	America	1	13	0.045	Asia	1	10	0.015	Oceania	1	10	0.045	/	/	/	/

Table S3. Statistical evaluation in the randomized alignments performed by each window detected as positive for structural RNA at intra-geographic lineages level. (L = Locus, W = Window, %FP = False positives rate)

Region	L	W	%Fp	Region	L	W	%Fp	Region	L	W	%Fp	Region	L	W	%Fp	Region	L	W	%Fp	Region	L	W	%Fp
Asia_cont	1	1	0.010	Asia_sure	1	3	0.015	Bra_Cl	1	7	0.025	Car_Cl	2	9	0.030	Col_Cl	2	5	0.055	Col_Cl	16	48	0.020
Asia_cont	1	2	0.030	Asia_sure	1	4	0.040	Bra_Cl	1	8	0.080	Car_Cl	2	10	0.025	Col_Cl	2	6	0.040	Col_Cl	16	49	0.040
Asia_cont	1	3	0.030	Asia_sure	1	5	0.030	Bra_Cl	1	9	0.025	Car_Cl	2	11	0.045	Col_Cl	2	7	0.030	Mex_Cl	1	7	0.045
Asia_cont	1	4	0.050	Asia_sure	1	6	0.045	Bra_Cl	1	10	0.040	Car_Cl	2	12	0.030	Col_Cl	2	8	0.050	Mex_Cl	1	8	0.040
Asia_cont	1	5	0.055	Asia_sure	1	7	0.010	Bra_Cl	1	11	0.035	Car_Cl	2	13	0.020	Col_Cl	2	9	0.035	Mex_Cl	1	9	0.030
Asia_cont	1	6	0.060	Asia_sure	1	8	0.020	Bra_Cl	1	12	0.030	Car_Cl	2	15	0.040	Col_Cl	2	10	0.045	Mex_Cl	1	10	0.035
Asia_cont	1	7	0.000	Asia_sure	1	9	0.010	Bra_Cl	1	13	0.060	Car_Cl	2	16	0.035	Col_Cl	2	11	0.045	Mex_Cl	1	11	0.045
Asia_cont	1	8	0.040	Asia_sure	1	10	0.030	Bra_Cl	1	15	0.065	Car_Cl	2	17	0.025	Col_Cl	2	12	0.045	Mex_Cl	1	12	0.030
Asia_cont	1	9	0.035	Asia_sure	1	11	0.025	Bra_Cl	1	16	0.030	Car_Cl	3	18	0.015	Col_Cl	2	13	0.020	Mex_Cl	1	13	0.020
Asia_cont	1	10	0.000	Asia_sure	1	12	0.040	Bra_Cl	1	17	0.075	Car_Cl	4	20	0.045	Col_Cl	2	14	0.045	Mex_Cl	1	14	0.045
Asia_cont	1	11	0.085	Asia_sure	1	13	0.030	Bra_Cl	2	20	0.045	Car_Cl	6	23	0.040	Col_Cl	2	15	0.025	Mex_Cl	1	15	0.030
Asia_cont	1	12	0.085	Asia_sure	2	14	0.030	Bra_Cl	3	23	0.035	Car_Cl	6	24	0.045	Col_Cl	2	16	0.070	Mex_Cl	1	16	0.030
Asia_cont	1	13	0.070	Asia_sure	3	15	0.010	Bra_Cl	5	26	0.030	Car_Cl	6	25	0.035	Col_Cl	4	20	0.045	Mex_Cl	1	17	0.025
Asia_cont	1	16	0.045	Asia_sure	4	16	0.035	Bra_Cl	6	27	0.045	Car_Cl	6	26	0.055	Col_Cl	4	22	0.030	Mex_Cl	2	19	0.045
Asia_cont	1	17	0.055	Asia_sure	5	17	0.020	Bra_Cl	8	32	0.045	Car_Cl	6	27	0.065	Col_Cl	6	25	0.020	Mex_Cl	4	22	0.025
Asia_cont	2	19	0.030	Asia_sure	7	19	0.015	Bra_Cl	11	37	0.015	Car_Cl	6	29	0.045	Col_Cl	7	26	0.030	Mex_Cl	6	25	0.015
Asia_cont	4	21	0.045	Asia_sure	8	20	0.025	Bra_Cl	14	42	0.045	Car_Cl	7	31	0.025	Col_Cl	7	27	0.040	Mex_Cl	6	26	0.035
Asia_cont	4	22	0.025	Asia_sure	8	21	0.030	Bra_Cl	14	43	0.045	Car_Cl	10	37	0.040	Col_Cl	7	28	0.650	Mex_Cl	6	27	0.030
Asia_cont	4	23	0.045	Asia_sure	10	24	0.045	Bra_Cl	14	44	0.030	Car_Cl	13	43	0.040	Col_Cl	7	29	0.040	Mex_Cl	6	28	0.060
Asia_cont	4	24	0.040	Asia_sure	10	25	0.025	Bra_Cl	15	47	0.045	Car_Cl	14	44	0.020	Col_Cl	7	30	0.020	Mex_Cl	6	29	0.030
Asia_cont	4	25	0.055	Asia_sure	10	28	0.050	Car_Cl	1	1	0.015	Car_Cl	15	45	0.025	Col_Cl	7	31	0.025	Mex_Cl	6	30	0.010
Asia_cont	7	30	0.045	Asia_sure	10	30	0.015	Car_Cl	2	2	0.015	Car_Cl	15	46	0.030	Col_Cl	8	32	0.065	Mex_Cl	8	35	0.035
Asia_cont	9	34	0.035	Bra_Cl	1	1	0.020	Car_Cl	2	3	0.020	Car_Cl	15	47	0.025	Col_Cl	10	36	0.055	Mex_Cl	9	38	0.045
Asia_cont	13	41	0.040	Bra_Cl	1	2	0.070	Car_Cl	2	4	0.030	Car_Cl	16	49	0.040	Col_Cl	10	37	0.025	Mex_Cl	12	44	0.035
Asia_cont	13	42	0.045	Bra_Cl	1	3	0.005	Car_Cl	2	5	0.090	Col_Cl	1	1	0.005	Col_Cl	12	41	0.025	Mex_Cl	12	45	0.025
Asia_cont	14	46	0.045	Bra_Cl	1	4	0.010	Car_Cl	2	6	0.030	Col_Cl	2	2	0.015	Col_Cl	15	45	0.030	Mex_Cl	12	46	0.010
Asia_sure	1	1	0.005	Bra_Cl	1	5	0.080	Car_Cl	2	7	0.040	Col_Cl	2	3	0.025	Col_Cl	15	46	0.045	Mex_Cl	13	48	0.040
